# Profiling neurotransmitter-evoked glial responses by RNA-sequencing analysis

**DOI:** 10.3389/fncir.2023.1252759

**Published:** 2023-08-14

**Authors:** Mengxiao Wang, Margaret S. Ho

**Affiliations:** School of Life Sciences and Technology, ShanghaiTech University, Shanghai, China

**Keywords:** glia, neurotransmitter, RNA-sequencing, metabolic suppression, signal transduction

## Abstract

Fundamental properties of neurons and glia are distinctively different. Neurons are excitable cells that transmit information, whereas glia have long been considered as passive bystanders. Recently, the concept of tripartite synapse is proposed that glia are structurally and functionally incorporated into the synapse, the basic unit of information processing in the brains. It has then become intriguing how glia actively communicate with the presynaptic and postsynaptic compartments to influence the signal transmission. Here we present a thorough analysis at the transcriptional level on how glia respond to different types of neurotransmitters. Adult fly glia were purified from brains incubated with different types of neurotransmitters *ex vivo*. Subsequent RNA-sequencing analyses reveal distinct and overlapping patterns for these transcriptomes. Whereas Acetylcholine (ACh) and Glutamate (Glu) more vigorously activate glial gene expression, GABA retains its inhibitory effect. All neurotransmitters fail to trigger a significant change in the expression of their synthesis enzymes, yet Glu triggers increased expression of neurotransmitter receptors including its own and nAChRs. Expressions of transporters for GABA and Glutamate are under diverse controls from DA, GABA, and Glu, suggesting that the evoked intracellular pathways by these neurotransmitters are interconnected. Furthermore, changes in the expression of genes involved in calcium signaling also functionally predict the change in the glial activity. Finally, neurotransmitters also trigger a general metabolic suppression in glia except the DA, which upregulates a number of genes involved in transporting nutrients and amino acids. Our findings fundamentally dissect the transcriptional change in glia facing neuronal challenges; these results provide insights on how glia and neurons crosstalk in a synaptic context and underlie the mechanism of brain function and behavior.

## Introduction

Neurons and glia are the two major cell types in the central nervous system (CNS). Whereas neurons are the major mediators for all aspects of brain function, glia, although abundantly seen, are usually considered passive bystanders that provide nutrient and support (Allen and Lyons, 2018; Kim et al., 2020; Perez-Catalan et al., 2021). Evolutionally, the percentage of glial cells increases with the complexity of organisms, from 16% in nematodes, 20% in *Drosophila*, 50% in mice, to 90% in human brains, suggesting that glia play a broader and more complex role (Verkhratsky, 2010; Zwarts et al., 2015; Losada-Perez, 2018; Yildirim et al., 2019; Doron and Goshen, 2020). Intriguingly, glial cells among these species are similar in the morphology and function. For instance, fly glia include cortex glia, astrocyte-like glia, ensheathing glia, and the surface glia (perineurial glia and subperineurial glia). Whereas the outermost perineurial glia (PNG) and subperineurial glia (SPG) are involved in forming the blood–brain barrier for protecting the nervous system, cortex glia, like mammalian astrocytes, are responsible to wrap around the cell bodies of neurons and provide them with nutrients. Astrocyte-like glia and ensheathing glia are both glia associated with neuropils, function similarly as mammalian astrocytes and oligodendrocytes, for mediating synapse formation, synaptic function, and axon insulation (Losada-Perez, 2018; Wilton et al., 2019; Yildirim et al., 2019; Sanuki, 2020; Hertenstein et al., 2021).

Lately, accumulating evidence has revealed new and exciting roles of glia, participating in the regulation of nervous system function in an active manner. In both *Drosophila* and mammals, glia have been shown as the third component in the tripartite synapse (Hillen et al., 2018; Durkee and Araque, 2019; Farizatto and Baldwin, 2023); they regulate synapse formation and synaptic transmission by responding to neurotransmitters, the chemical molecules that convey signals after releasing into the synaptic cleft. In nanoscale, neurotransmitters are released to activate the corresponding receptors on the postsynaptic membrane, propagating the signaling relay from the presynaptic to the postsynaptic compartment. Like postsynaptic compartments, glia, also express receptors on the membranes that respond to these neurotransmitters. In addition, glia express transporters which uptake and recycle extra neurotransmitters released into the cleft. Thus, it is crucial to consider how glia respond to neurotransmitters and incorporate these effects when analyzing the synaptic transmission for larger contexts.

Neurons secrete a variety of neurotransmitters, including acetylcholine (ACh), gamma-aminobutyric acid (GABA), glutamate (Glu), dopamine (DA), adenosine triphosphate (ATP), serotonin (5-HT), opioids, endocannabinoids, and etc. (Deng et al., 2019; Vogt, 2019; Cox et al., 2020; De Deurwaerdere and Di Giovanni, 2020; Kilb and Kirischuk, 2022; Wei et al., 2022; Wiseman, 2022; Suwara et al., 2023). These neurotransmitters, some also released by glia, convey activation or inhibitory signals. Here we developed a protocol purifying glia from the adult fly brains *ex vivo*. By RNA-sequencing analysis, we found that incubation with different types of neurotransmitters triggers differential gene expressions in glia. Distinct and overlapping patterns of these transcriptomes were identified, implicating that different types of neurotransmitters induce unique signature patterns in gene expression; interconnected crosstalk among them also occurred at the transcriptional level. A general metabolic suppression in glia was also evoked by all types of neurotransmitters except the DA, which upregulates a number of genes involved in transporting nutrients and amino acids.

## Results

### Purification and RNA-sequencing analysis of adult fly glia *ex vivo*

**FIGURE 1:**
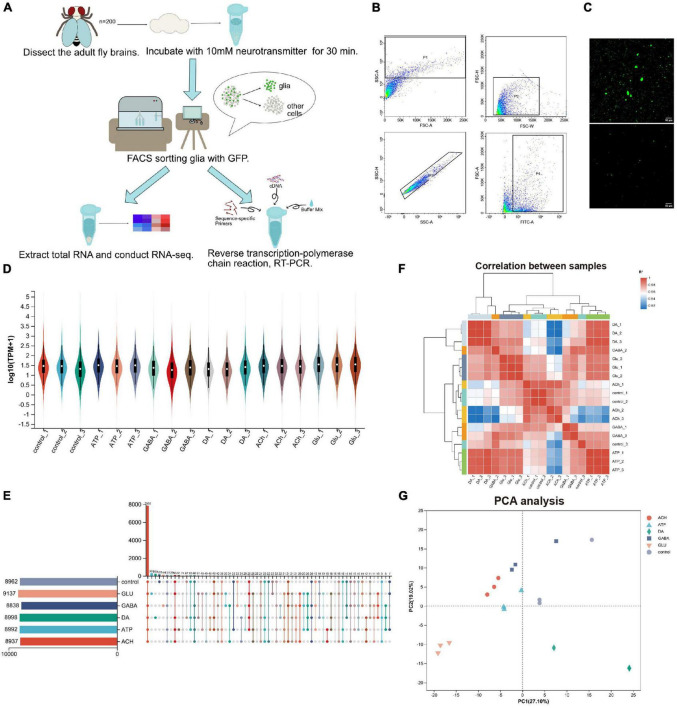
Purification and RNA-sequencing of neurotransmitter-treated adult fly glia. **(A)** An schematic diagram illustrating the purification, sorting, and gene expression analysis of adult fly glia treated with neurotransmitters *ex vivo*. A total of 200 adult fly brains were dissected and treated with different neurotransmitters for 30 min. Neurotransmitters used: Glu, DA, ATP, ACh, and GABA. The brain tissues were homogenized into suspended and GFP-positive single cells and subsequently sorted by FACS. Novaseq sequencing technology was used to sequence all mRNAs in the transcriptome, and Illumina Truseq™ RNA sample prep Kit was used to construct the library. The sequencing results were verified by RT-qPCR. **(B)** The FACS sorting plot of adult fly glia. P4 was classified according to the fluorescence intensity, and a FITC-A value over 10^2^ was identified as GFP-positive single-cell population. **(C)** Confocal microscopy visualizing the cell suspension before and after FACS sorting. Scale bar, 50 μm. **(D)** The Violin plot of sample expression distribution. Each color represents one sample, and the enlarged part represents the region with the most concentrated gene expression. **(E)** The Upset plot of sample expression distribution. The horizontal bar chart on the left represents the expression quantity of each sample. The middle matrix shows the number of overlapping genes in each category. **(F)** Sample distance heatmap (Pearson correlation analysis). The correlation analysis indicates that the results from three replicates are consistent and the data are reliable (ideal biological replication *R*^2^ > 0.92). **(G)** Principal component analysis (PCA analysis). After dimensionality reduction analysis, the samples have relative coordinate points on the principal component, indicating the distance among the samples. The closer the distance, the higher the similarity between the samples.

To investigate how glia respond to neurotransmitters, we developed a protocol analyzing gene expression in adult fly glia (Figure 1A). Adult fly brains expressing a membranous GFP (*UAS-mCD8GFP*) under the control of a pan-glial driver *repo-GAL4* (*repo > mCD8GFP*) were dissected and incubated with different types of neurotransmitters (10 mM) for 30 min. The neurotransmitters we used include: Glu, DA, ATP, ACh, and GABA. GFP-positive adult fly glia were purified by the fluorescence activated cell sorting (FACS) and total RNAs were extracted from these glia for further sequencing analysis. Expression changes of genes of interest were verified again by the reverse transcription quantitative PCR (RT-qPCR). Due to the heterogeneity in the size and shape of glial cells, adult fly brain cells were suspended into single cells and sorted by the criteria whether the cells express GFP (Figure 1B). Interestingly, the sorting plot revealed that 8–10% of total cells were GFP-positive, correlating with the reported number of glia in adult fly brains (Croset et al., 2018; Brunet Avalos et al., 2019; Park et al., 2022). After removal of cell debris, single GFP-positive glial cells were visualized by confocal microscopy (Figure 1C).

By RNA-sequencing analysis, we were able to analyze the glial response at the transcription level to each type of neurotransmitters; the differences in individual gene expression were compared. A total of 720 million reads of sequencing samples were obtained, with each transcriptome containing about 0.4 million reads in three biological replicates. A total of 16,107 genes were detected including 2,631 uncharacterized genes (annotated in CG number). Total 46,048 expressed transcripts including 18,763 new transcripts were identified. RSEM software was used to quantitatively analyze the expression level of transcripts, and TPM conversion was performed to homogenize the sequencing length and depth of transcripts, so to obtain standardized expression level of transcripts. According to the Violin plot showing the sample expression distribution, the gene expression level in each transcriptome is similar, ensuring the same amount of information collected from different transcriptomes (Figure 1D). In addition, about 9,000 genes were identified in each transcriptome as revealed by the sample expression distribution Upset plot, with over 7,800 overlapping genes identified in all six groups and the rest expressing in different groups (Figure 1E). Sample distance heatmap (Figure 1F) and PCA analysis (Figure 1G) revealed a strong correlation between the bio-replicated samples for each group treated with different neurotransmitters. These results suggest that the difference between replicates of each group is relatively large, whereas among replicates themselves is small. The RNA-sequencing results are therefore reliable, consistent, and qualified.

### Identification of differentially expressed genes in neurotransmitter-treated adult fly glia

**FIGURE 2:**
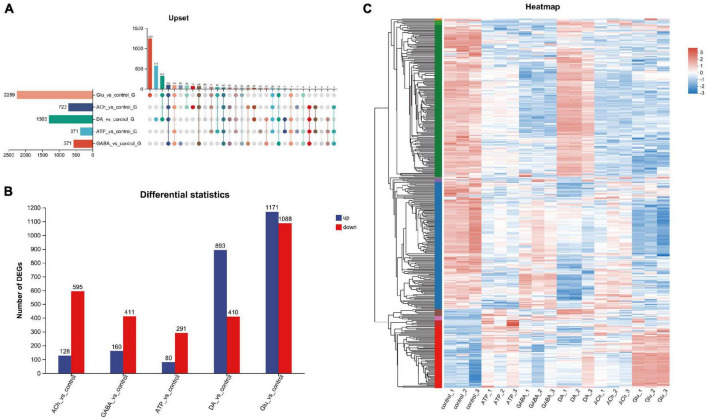
Analysis of differentially expressed genes (DEGs) in neurotransmitter-treated adult fly glia. **(A)** The Upset plot of DEGs. The horizontal bar chart on the left represents the number of differential genes expressed in each sample. The matrix revealed the number of overlapping genes in each category. **(B)** Bar graph showing the number of DEGs in each group. The number of upregulated genes (blue) and the downregulated genes (red) were listed on the top of the bars for each group. Note that more upregulated than the downregulated genes were identified for DA- and Glu-treated adult fly glia, whereas the rest is the opposite. Down-regulated genes were identified as FC ≤ 0.5 and up-regulated genes as FC ≥ 2. Adj. *p*-value ≤ 0.05. FC, fold change; adj., adjusted (Benjamin–Hochberg multiple comparison method). **(C)** Differential gene heatmap comparing glia treated with different types of neurotransmitters. *Y*-axis: individual genes categorized into groups with left color labels green, blue, and red. *X*-axis: five different sample groups. A color tile is present on the right with red and blue indicate higher or lower expression level of the designated genes, respectively. The left *Y*-axis also reveals the tree diagram of gene clustering and the module diagram of sub clustering, *Z*-score is used for standardization, and the value of TPM +1 is converted by log10. When the score is lower than the average value, *z* is negative; otherwise, it is positive.

Next, genes differentially expressed among these groups were analyzed. Gene set analysis was carried out and the Upset plot revealed the total number of differentially expressed genes (DEGs) in adult fly glia upon neurotransmitter addition. A total of 2,259 DEGs were identified in Glu-treated glia, whereas only 371 DEGs were detected in ATP-treated glia (Figure 2A). Among these identified DEGs, 1,171 upregulated and 1,088 downregulated genes were identified in Glu-treated glia, whereas only 80 upregulated and 291 downregulated genes were identified in ATP-treated glia (Figure 2B). As more upregulated genes were identified in the Glu and DA groups, more downregulated genes were detected in the ACh, GABA, and ATP groups (Figure 2B). These results suggest that neurotransmitters have differential effects on the glial gene expression. A heatmap of gene cluster analysis was drawn to compare the expression level of individual gene in each group (Figure 2C). *Z* score was used to standardize the gene expression: when the score is lower than the average value, *z* is negative; otherwise, it is positive. As shown in the heatmap, genes in the top panel (green label on the left) were mostly upregulated in the DA-treated glia compared to the control group, whereas the same set of genes were mostly downregulated in the GABA-, ACh-, or Glu-treated glia. Genes in the middle panel (blue label on the left) were mostly downregulated in glia treated with all five types of neurotransmitters. At last, genes in the bottom panel (red label on the left) exhibited a low expression in the control group, yet mostly upregulated in the Glu-treated glia (Figure 2C). Consistently, changes in some of these genes involved in signal transduction, metabolism, and function of glial cells were also verified by RT-qPCR in our hand (Supplementary Figures 1, 2).

On the other hand, DEGs categorized by different analyses revealed further information on the function of affected genes (Supplementary Figures 3–5). For instance, COG classification showed that most genes affected in all five groups of glia were poorly characterized (Group S), revealing unrecognized glial cell functions that remain to be investigated. GO annotation and KEGG analysis showed similar trends in the number of genes involved in different cellular processes or pathways for all five types of neurotransmitter-treated glia. Considering that neurotransmitters likely regulate glia in similar mechanisms, it is feasible that no dramatic change in one single type of cellular events is detected.

### Neurotransmitters evoke changes in the expression of related receptors and transporters in adult fly glia

Despite that glia are not excitable cells, they utilize similar repertoires of synthesis enzymes, receptors, and transporters when transmitting the cellular signals in response to neurotransmitters (Fellin and Carmignoto, 2004; Fiacco and McCarthy, 2006; Hartl et al., 2007; de Tredern et al., 2021). Next, we analyzed the expression change in a list of genes characterized for their function in the synthesis, transport, and receptor-based signaling of neurotransmitters. Genes selected are the synthesis enzymes, transporters, and receptors for the five types of neurotransmitters and they are listed in Table 1.

**TABLE 1:** A list of genes involved in the synthesis, transport, and receptor-based signaling of neurotransmitters.

Function	Gene ID	Gene	ACh	ATP	DA	GABA	Glu
Synthesis	FBgn0000303	ChAT					
	FBgn0013672	mt:ATPase6					
	FBgn0019644	ATPsynB					
	FBgn0039830	ATPsynC					
	FBgn0016120	ATPsynD					
	FBgn0038224	ATPsynE					
	FBgn0035032	ATPsynF					
	FBgn0010612	ATPsynG					**−**
	FBgn0016119	ATPsynCF6					
	FBgn0016691	ATPsynO					
	FBgn0011211	blw					
	FBgn0010217	ATPsynbeta					
	FBgn0020235	ATPsyngamma					
	FBgn0028342	ATPsyndelta					
	FBgn0004516	Gad1	**+**		**+**		**+**
	FBgn0000422	Ddc					
	FBgn0001098	Gdh					
	FBgn0036663	CG9674					
Transporters	FBgn0270928	VAChT					**+**
	FBgn0005775	Con					**+**
	FBgn0052103	SCaMC					
	FBgn0033911	VGAT					
	FBgn0039915	Gat	**−**		**+**	**−**	**−**
	FBgn0034136	DAT					
	FBgn0260964	Vmat			**+**		
	FBgn0031424	VGlut					
	FBgn0010497	dmGlut					
	FBgn0026439	Eaat1			**+**	**−**	
	FBgn0026438	Eaat2					
Receptors	FBgn0000036	nAChRalpha1			**+**		**+**
	FBgn0000039	nAChRalpha2					
	FBgn0015519	nAChRalpha3					**+**
	FBgn0266347	nAChRalpha4					**+**
	FBgn0028875	nAChRalpha5	**+**	**+**	**+**	**+**	**+**
	FBgn0032151	nAChRalpha6	**+**	**+**	**+**	**+**	**+**
	FBgn0086778	nAChRalpha7	**+**	**+**	**+**	**+**	**+**
	FBgn0000038	nAChRbeta1					**+**
	FBgn0004118	nAChRbeta2					
	FBgn0031261	nAChRbeta3					**−**
	FBgn0000037	mAChR-A					**+**
	FBgn0037546	mAChR-B					**+**
	FBgn0029909	mAChR-C					
	FBgn0039747	AdoR			**+**		**−**
	FBgn0035538	DopEcR				**+**	**+**
	FBgn0011582	Dop1R1					**+**
	FBgn0266137	Dop1R2					
	FBgn0053517	Dop2R					**+**
	FBgn0260446	GABA-B-R1			**+**		**+**
	FBgn0027575	GABA-B-R2					
	FBgn0031275	GABA-B-R3					**+**
	FBgn0019985	mGluR		**+**	**+**		**+**
	FBgn0004619	GluRIA					
	FBgn0264000	GluRIB					**+**
	FBgn0004620	GluRIIA	**−**	**−**		**−**	**−**
	FBgn0020429	GluRIIB					
	FBgn0046113	GluRIIC					
	FBgn0028422	GluRIID			●	●	
	FBgn0051201	GluRIIE			**−**		**−**
	FBgn0262869	Gfrl					**+**

The changes in the expression of these genes are denoted by “+” (increased expression), “**−**” (decreased expression), “ ” (no change), and “●” (not tested) upon adding different types of neurotransmitters.

**FIGURE 3:**
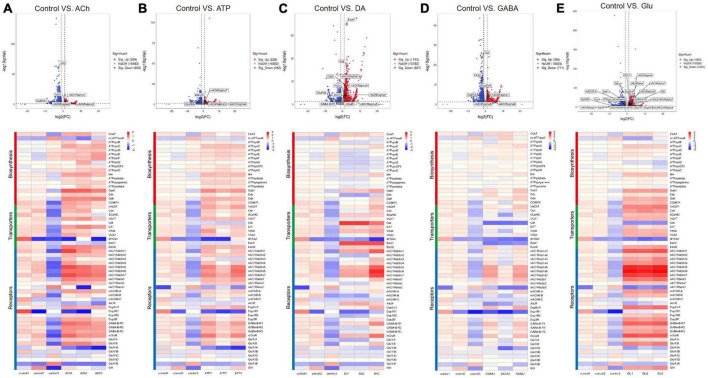
Neurotransmitters trigger changes in the expression of related receptors and transporters in glia. **(A–E)** The Volcano plot (top) and the cluster heatmap (bottom) are shown. Genes shown in both are similarly selected by their expression level change with the adjusted *p*-value < 0.05, log2FC > 1 for the increased gene expression (red); the adjusted *p*-value < 0.05, log2FC < –1 for the decreased gene expression (blue). Multiple comparisons were conducted by the Benjamin–Hochberg analysis. DEGs are boxed out in the Volcano plot. Panels **(A–E)** represent analysis for glia treated with ACh **(A)**, ATP **(B)**, DA **(C)**, GABA **(D)**, and Glu **(E)**.

As shown by the Volcano plot and heatmap in Figure 3, significant changes in the expression of genes listed in Table 1 were uncovered. We used “+” or “−” symbol for indicating genes with increased expression (the adjusted *p*-value < 0.05, log2FC > 1) or decreased expression (the adjusted *p*-value < 0.05, log2FC < −1), respectively in Table 1. A color tile along with the number designation was shown in the upper-right corner of the heatmap in Figure 3 for indicating the difference in gene expression. By default, a three color scale is used with blue indicating low expression values (2–4-fold lower), white indicating similarly expressed genes, and red representing highly expressed genes (2–4-fold higher). Genes with expression differentially affected by the neurotransmitters were selected and shown in the Volcano plot. Overall, neurotransmitters failed to evoke significant change in the expression of synthesis enzymes and triggered more change in the receptor expression. Among the five types of neurotransmitters, Glu activate the expression of Table 1 genes more significantly. Whereas Glu mainly evoked upregulation in receptor expression, GABA rarely caused any increase, correlating with its inhibitory function (Figures 3D, E).

Specifically, ACh increased the expression of *glutamate decarboxylase 1* (*GAD1*) and *nicotinic acetylcholine receptor alpha 1, 5-7* (*nAChRα1, 5-7*), yet decreased the expression of *Glutamate receptor IIA* (*GluRIIA*) (Control vs. ACh, Figure 3A). ATP also decreased the *GluRIIA* expression, and increased the expression of *nAChRα1, 5-7* as potently as ACh (Control vs. ATP, Figure 3B). DA upregulated the GABA transporter *GAT* and the *Excitatory amino acid transporter 1* (*Eaat1*), both are transporters for neurotransmitter trafficking (Control vs. DA, Figure 3C). Whereas GABA did not elicit a dramatic change in the overall gene expression, Glu elicited increased expression in *nAChRα1, 5-7*, *Dopamine/Ecdysteroid receptor* (*DopEcR*), *metabotropic GABA-B receptor subtype 1* (*GABA-B-R1*), *metabotropic Glutamate Receptor (mGluR)*, and *Glutamate receptor IB* (*GluRIB*) (Figure 3E).

### Neurotransmitters trigger changes in the expression of genes dictated for glial function

**FIGURE 4:**
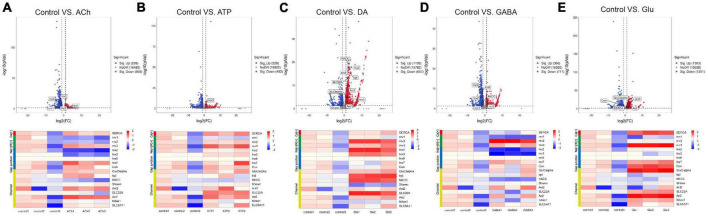
Neurotransmitters trigger changes in the expression of genes related to glial activity. The Volcano plot (top) and the cluster heatmap (bottom) are shown. Genes shown in both are similarly selected by their expression level change with the adjusted *p*-value < 0.05, log2FC > 1 for the increased gene expression (red); the adjusted *p*-value < 0.05, log2FC < –1 for the decreased gene expression (blue). Multiple comparisons were conducted by the Benjamin–Hochberg analysis. DEGs are boxed out in the Volcano plot. Panels **(A–E)** represent analysis for glia treated with ACh **(A)**, ATP **(B)**, DA **(C)**, GABA **(D)**, and Glu **(E)**.

It has been widely recognized that glia are part of the tripartite synapse and respond to neuronal signals by calcium wave propagation, a functional way to relay signals to adjacent or distal glia via gap junctions (Kielian and Esen, 2004; Deitmer and Rose, 2010; Verkhratsky, 2010; De Bock et al., 2013; Mu et al., 2019; Spray et al., 2019). To further investigate whether neurotransmitters trigger change in genes involved in these aspects, a list of genes encoding channels, transporters, and gap junctions were compiled and analyzed (Table 2). Volcano plot and heatmap were also illustrated in Figure 4, with changes in the expression level of these genes described similarly in Table 1 and Figure 3. Interestingly, expressions of most genes in these categories were upregulated upon ATP, DA, or Glu addition, whereas some inhibitory effects were seen in the group of ACh- and GABA-treated glia. These results suggest that in general neurotransmitters trigger glial activation.

**TABLE 2:** A list of genes involved in glial calcium activity.

Function	Gene ID	Gene	ACh	ATP	DA	GABA	Glu
Glial function	FBgn0263006	SERCA					**+**
	FBgn0015776	nrv1	**−**			**−**	
	FBgn0015777	nrv2			**+**	**−**	
	FBgn0032946	nrv3		**+**	**+**	**+**	**+**
	FBgn0027108	Inx2	**−**		**+**	**−**	
	FBgn0265274	Inx3	**−**		**+**	**−**	
	FBgn0027107	Inx6	●	●	●		●
	FBgn0027106	Inx7	**+**				
	FBgn0005775	Con					**+**
	FBgn0024963	GluClalpha			**+**		**+**
	FBgn0005614	trpl			**+**		
	FBgn0051547	NKCC			**+**	**−**	
	FBgn0031039	Shawn			**−**		
	FBgn0025111	Ant2					
	FBgn0037140	SLC22A			**+**		
	FBgn0036043	Ae2	**+**		**+**		**+**
	FBgn0259111	Ndae1					
	FBgn0031998	SLC5A11			**+**		

These genes encode the proteins that function as calcium channel, gap junction, or transporter. The changes in the expression of these genes are denoted by “+” (increased expression), “**−**” (decreased expression), “ ” (no change), and “●” (not tested) upon adding different types of neurotransmitters.

Specifically, the expression of *Sarco/endoplasmic reticulum Ca(2+)-ATPase* (*SERCA*) was upregulated in the Glu-treated glia, suggesting that Glu triggers glial calcium activity. The expression of *nervana 3* (*nrv3*) was upregulated in ATP- and Glu-treated glia, whereas *nervana 2* (*nrv2*) was more highly expressed in the DA and GABA group. For gap junction genes, *Innexin 2* (*Inx2*) and *Innexin 3* (*Inx3*) were both upregulated in the DA-treated glia, yet downregulated in ACh- and GABA-treated glia. Moreover, genes encoding channels such as *Glutamate-gated chloride channel subunit alpha* (*GluClalpha*) and *Ae2* in Glu group, and *transient receptor potential-like* (*trpl*), *sodium potassium chloride cotransporter* (*NKCC*) and *SLC22A family member* (*SLC22A*) in the DA group were more highly expressed. On the other hand, the expressions of *Shawn* and *NKCC* were downregulated in the DA- or ACh- and GABA-treated glia, respectively (Figure 4C). Taken together, these results suggest that neurotransmitters trigger changes in the expression of genes functioning in glial activity.

### Neurotransmitters elicit metabolic changes in adult fly glia

**FIGURE 5:**
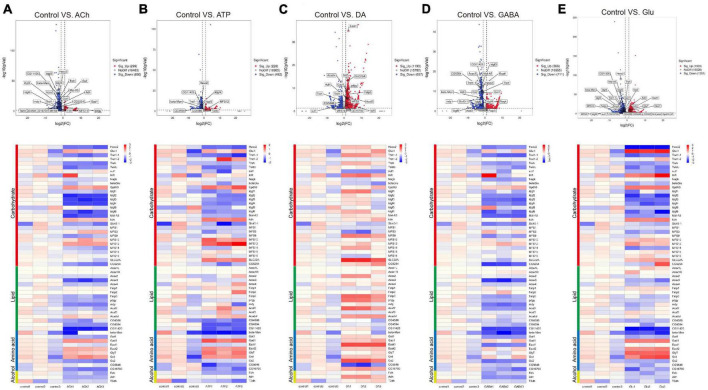
Neurotransmitters trigger changes in the expression of genes related to metabolism. **(A–E)** The Volcano plot (top) and the cluster heatmap (bottom) are shown. Genes shown in both are similarly selected by their expression level change with the adjusted *p*-value < 0.05, log2FC > 1 for the increased gene expression (red); the adjusted *p*-value < 0.05, log2FC < –1 for the decreased gene expression (blue). Multiple comparisons were conducted by the Benjamin–Hochberg analysis. DEGs are boxed out in the Volcano plot. Panels **(A–E)** represent analysis for glia treated with ACh **(A)**, ATP **(B)**, DA **(C)**, GABA **(D)**, and Glu **(E)**.

Accumulating evidence has indicated that metabolic changes in neurons associate with brain dysfunction (Mlody et al., 2016; Diaz-Garcia et al., 2017; Onkar et al., 2020; Rickman et al., 2020; Bonvento and Bolanos, 2021; Li et al., 2021). On the other hand, not a lot has been explored for how glia react to neuronal challenges from a metabolic view. Here we investigated metabolic genes differentially expressed in adult fly glia upon adding neurotransmitters. Searches done using the COG, GO, and KEGG databases identified genes in four types of metabolism including carbohydrates, lipid, amino acid, and alcohol (Table 3). Volcano plot and heatmap were also illustrated in Figure 5, with changes in the expression level of these genes described similarly in Table 1 and Figure 3. Overall, expressions of these metabolic genes were mostly downregulated in ACh- or GABA-treated glia, whereas ATP and Glu also evoked suppression for most genes but with some exception. These results suggest that metabolic suppression might be a general event when glia are in the presence of neurotransmitters. Interestingly, DA triggered increased expression for most metabolic genes, implicating a different response pathway for glia upon DA addition.

**TABLE 3:** A list of genes involved in metabolism.

Function	Gene ID	Gene	ACh	ATP	DA	GABA	Glu
Carbohydrate metabolism	FBgn0041629	Hexo2	**−**	**−**		**−**	**−**
	FBgn0264574	Glut1					
	FBgn0050035	Tret1-1	**−**	**−**	**+**	**−**	**−**
	FBgn0033644	Tret1-2					
	FBgn0003748	Treh	**−**		**+**	**−**	**−**
	FBgn0023477	Taldo	**−**	**−**			**−**
	FBgn0028563	sut1			**−**		**−**
	FBgn0283499	InR			**+**		**+**
	FBgn0038321	Nagk				**−**	
	FBgn0270927	betaGlu					**−**
	FBgn0263048	Gpdh3					**+**
	FBgn0020416	Idgf1					
	FBgn0020415	Idgf2	**−**			**−**	**−**
	FBgn0020414	Idgf3	**−**	**−**		**−**	**−**
	FBgn0026415	Idgf4	**−**	**−**		**−**	**−**
	FBgn0064237	Idgf5					
	FBgn0013763	Idgf6	**−**			**−**	**−**
	FBgn0050359	Mal-A5	**−**			**−**	**−**
	FBgn0261955	kdn					**+**
	FBgn0035968	Slc45-1				**−**	
	FBgn0050272	MFS1					
	FBgn0031307	MFS3					**−**
	FBgn0038799	MFS9			**+**	**−**	
	FBgn0030452	MFS10					
	FBgn0033234	MFS12		**+**			
	FBgn0010651	MFS14	**−**		**+**		**−**
	FBgn0034392	MFS15					
	FBgn0034611	MFS16					
	FBgn0037140	SLC22A			**+**		
	FBgn0040383	CG5254	**−**			**−**	**−**
Lipid metabolism	FBgn0038730	Acsx1L	●	●	●	●	
	FBgn0038731	Acsx1R	●	●			
	FBgn0038732	Acsx2					
	FBgn0038733	Acsx3	**−**			**−**	**−**
	FBgn0038734	Acsx4					
	FBgn0267828	Fatp1					
	FBgn0265187	Fatp2					
	FBgn0034999	Fatp3			**+**	**−**	
	FBgn0027601	pdgy	**−**		**+**		
	FBgn0004797	mdy	**−**			**−**	
	FBgn0031703	Acsf2			**+**	**−**	
	FBgn0029945	Acsf3					
	FBgn0034432	Acadvl			**+**		
	FBgn0029924	CG4586		**−**			**−**
	FBgn0032161	CG4594	**−**	**−**			
	FBgn0037166	CG11426	**−**	**−**		**−**	
Amino acid metabolism	FBgn0037215	beta-Man	**−**	**−**		**−**	**−**
	FBgn0001124	Got1	**−**			**−**	**−**
	FBgn0004516	Gad1	**+**		**+**		**+**
	FBgn0026439	Eaat1			**+**	**−**	
	FBgn0026438	Eaat2					
	FBgn0034911	GlyT					**+**
	FBgn0001134	Grd					**+**
	FBgn0001145	Gs2	**−**		**+**	**−**	**−**
	FBgn0039525	CG5646			**−**		**−**
	FBgn0030816	CG16700					
Alcohol metabolism	FBgn0011768	Fdh	**−**				**−**
	FBgn0000055	Adh	**−**		**+**	**−**	**−**
	FBgn0017482	T3dh	**−**		**+**		**−**

Genes are categorized into metabolism related to carbohydrates, lipid, amino acid, and alcohol. The changes in the expression of these genes are denoted by “+” (increased expression), “**−**” (decreased expression), “ ” (no change), and “●” (not tested) upon adding different types of neurotransmitters.

Specifically, expressions of the metabolic genes including *Hexosaminidase 2 (Hexo2), Trehalose transporter 1-1 (Tret1-1)*, and *Imaginal disc growth factor 2-4, 6* (*Idgf2-4, 6*) in the Carbohydrate section were downregulated in the ACh- and GABA-treated glia. The expressions of *Acyl-CoA synthetase X3* (*Acsx3*) and a putative lipid phosphatase *CG11426* in the Lipid section were downregulated in ACh- and GABA-treated glia, while the expression of *CG5254*, a putative α-ketoglutarate transporter, was particularly affected by GABA (Figures 5A, D). These results indicate that the DEGs identified for ACh- and GABA-treated glia are similar.

On the other hand, a mixture of upregulated and downregulated genes were identified in the ATP- and Glu-treated glia. For the Carbohydrate section, the expressions of *Glycerol-3-phosphate dehydrogenase 3* (*Gpdh3*), *knockdown* (*kdn*), *Major Facilitator Superfamily Transporter 12* (*MFS12*), and *SLC22A family member* (*SLC22A*) were upregulated in both types of glia, although in different degrees. The expressions of glucose transporter *Glut1* and *Insulin-like receptor* (*InR*) were specifically upregulated in Glu-treated glia. The expressions of putative lipid-related genes like *CG4586*, *CG4594*, and *CG11426* were downregulated in both types of glia, with the expression of *CG11426* most affected in the Glu-treated glia. In the Amino acid section, the expressions of *Gad1*, *Eaat2*, *Glycine transporter* (*GlyT*), and *Glycine receptor* (*Grd*) were upregulated in both ATP- and Glu-treated glia (Figures 5B, E).

In the DA-treated glia, most metabolic genes were upregulated in all four types of metabolism groups. The most affected genes included *Major Facilitator Superfamily Transporter 9* (*MFS9*) and *SLC22A* in the Carbohydrate section; *Fatty acid transport protein 3* (*Fatp3*), *pudgy (pdgy)*, *Acyl-CoA synthetase family member 2* (*Acsf2*), and *Acyl-CoA dehydrogenase very long chain* (*Acadvl*) in the Lipid section; *Eaat1* and *Gs2* in the Amino acid section. Interestingly, *sugar transporter 1* (*sut1*) and the putative L-arginine *solute carrier family 25 member 45* (*SLC25A45*, *CG5646*) were specifically downregulated in the presence of DA (Figure 5C). Taken together, these results suggest that metabolic suppression in glia is a general event upon neurotransmitter challenges except for DA.

## Discussion

In the present study, we profiled the gene expression change in adult fly glia in the presence of different neurotransmitters. It is widely recognized that glia are the central elements in the tripartite synapse. They express the corresponding receptors, respond to neurotransmitters, mediate their uptake and recycling, and relay functional changes downstream. Thus, our findings provide both an overview on the gene expression change at the transcription level and implications on specific cellular events that orchestrate these glial responses. Analyses using different methods and qPCR results have also validated our RNA-sequencing results.

**FIGURE 6:**
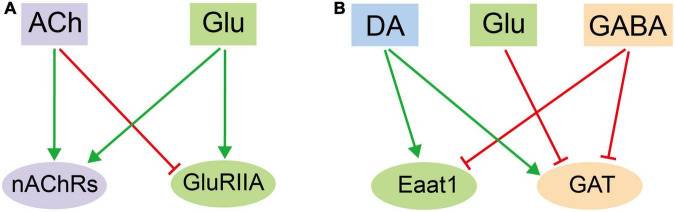
An illustration on the mutual regulatory network in adult fly glia among different neurotransmitters. **(A)** The reciprocal effect of ACh and Glu on glia by regulating the receptor expression. **(B)** The interactive and overlapping network of neurotransmitter-triggered glial responses among DA, Glu, and GABA. Note that the three exhibit reciprocal effect on one or another.

It is plausible to hypothesize that different types of neurotransmitters induces different cellular responses in glia. Thus, the overall profile of DEGs for each transcriptome should be distinct and separate from each other. Nonetheless, we identified profiles overlapping in number, pattern, and identity. ACh and Glu treatment both upregulate the *nAChRs* expression, suggesting that these receptors augment their responses by increasing their availability on the glial surface for receiving ACh. While both ACh and Glu increase the receptor expression of their own: Glu increases the expression of *nAChRs*, yet ACh decreases the expression of *GluRIIA*, suggesting a reciprocal effect of ACh and Glu on glia (Figure 6A). Furthermore, DA increases the expressions of *GAT* and *Eaat1*, transporter for GABA and Glu, respectively, whereas Glu decreases the *GAT* expression. These results suggest opposite effects of DA and Glu on the GABA-mediated signaling pathway. In contrast, GABA decreases the *Eaat1* expression, indicating that DA and GABA also regulate Glu transport in opposite means. On the other hand, GABA inhibits its own transport by decreasing the *GAT* expression, suggesting a potential feedback loop on controlling the GABA availability. These results together point to an interactive and overlapping network of neurotransmitter-triggered glial responses (Figure 6B). Our results demonstrate the complexity in the interactive network at the transcription level. Of note, all five types of neurotransmitters fail to evoke a dramatic change in the expression of genes encoding synthesis enzymes for neurotransmitters, suggesting that glia do not respond to neurotransmitters by increasing their amounts, but to allocate the existing available signaling molecules for their trafficking and release, so to efficiently orchestrate the glial responses. Notably, the change in the gene expression also reflects a potential modulation on glial function. To this end, genes involved in propagating the glial calcium waves are also differentially expressed upon adding different types of neurotransmitters.

Recently, metabolic changes have emerged as critical mechanisms associated with brain dysfunction. A lot of observations have been made in neurons, whereas metabolic changes in glia in the context of synapse activation and neuronal function have not been largely explored. Our findings reveal that metabolic suppression in glia might be a general event upon neurotransmitter challenge. The expression of most metabolic genes in glia are suppressed when challenged with most types of neurotransmitters. Notably, DA triggers a general increase in the expression of metabolic genes including the choline transporter *SLC22A*, suggesting that DA acts differently from other types of neurotransmitters in regulating glial metabolism in the context of synaptic transmission. It has been shown that glia express a wide range of solute carrier family transporters that transport sugar, nutrients, amino acid, etc. (Visser et al., 2007; Awasaki et al., 2008; Zhu et al., 2008; Boudko, 2012; Liu et al., 2015; Parkhurst et al., 2018; McMullen et al., 2021; De Backer and Kadow, 2022). In our study, neurotransmitters trigger changes in the expression of different *SLC* genes, implicating a role for SLC transporters in transducing neurotransmitter-triggered metabolic changes in glia.

Glia are the central elements of tripartite synapse and have long been recognized for their pivotal function in regulating a plethora of cellular processes underlying brain function and behavior. The present study provides insights on how glia respond to neurotransmitters at the transcription level. These findings illustrate a complex transcriptional network that includes characterized genes and the ones await to be further analyzed. Future work will be required to uncover how these DEGs uniquely relay the neurotransmitter-evoked signals in glia, so to construct the foundation underlying the mechanism of glia-neuron crosstalk in a synaptic context.

## Materials and methods

### Fly stock and husbandry

Flies were raised on a fly food (10 g agar, 52.5 g glucose, 60 g dry yeast, 59.1 g corn flour, 26.2 g brown sugar, 10.1 ml antiputrefactive #1 (418 ml propionic acid, 41.5 ml 85% phosphoric acid, and 540.5 ml ddH_2_O), and 12.5 ml antiputrefactive #2 (200 g methyl p-hydroxybenzoate add to 95% ethanol to 1 L) in a 12/12 h light/dark condition at 25°C with 70% humidity. All strains were obtained from Bloomington Stock Center, the Vienna *Drosophila* RNAi Center (VDRC), or as gifts from colleagues. The following stocks were used: *repo-GAL4*, *UAS-mCD8GFP*, *UAS-mCD8GFP; repo-GAL4*, *UAS-GCamp6.0*, *UAS-Gcamp6.0; repo-Gal4*.

### Adult fly brain dissociation and cell collection

The flies used to sort glia were kept in incubators at 25°C, 70% humidity, 12/12 h light/dark condition, for 2∼3 days after eclosion. The brains expressing *UAS-mCD8GFP; repo-GAL4* were dissected and dissociated as described (Croset et al., 2018; Li et al., 2022; Lu et al., 2023). Male and female adult flies of 100 each were dissected in cold Schneider’s *Drosophila* medium (SDM: Gibco) and immediately transferred to 1 ml cold SDM with 10 mM of different neurotransmitters added. Neurotransmitters used: acetylcholine chloride (Sigma A6625-25G), dopamine hydrochloride (MCE HY-B0451A), adenosine 5′-triphosphate disodium salt hydrate (Sigma A2383), γ-aminobutyric acid (MCE HY-N0067), and glutamate (Sigma G0355000). After incubated at room temperature on a shaker for 30 min, the brains were washed three times with 1 ml SDM for 10 min each time, then once again with 1XPBS and incubated in 300 μl SDM containing 100 U/ml papain (MCE, HY-P1645) and 2.5 mg/ml collagenase TL (Roche, 5401020001) for 30 min. After incubation for 5 min, the solution was mixed by pipets and centrifuged at 1,500 rpm for another 5 min for three times. A total of 400 μl cold SDM was added to inactivate enzyme. The supernatant was then filtered with 40 μm Cell strainer (Thermo/Fisher, T_70122363547), centrifuged at 400 *g*, 4°C for 7 min, then re-suspended with 500 μl SDM in a flow tube. Single-cell suspensions were screened by the BD FACS Aria Fusion Cell Sorter flow cytometry and sorted using a 100 μm nozzle. Flow-sorted glia were collected with 500 μl TRIzol Reagent (Thermo, 15596026) in the RNase-free centrifuge tubes. Average 30,000 glial cells were collected in each centrifuge tube and stored in liquid nitrogen.

### RNA extraction

Total RNA was extracted from the purified glia using TRIZOL and Viral DNA/RNA Mini Purification Kit (MCE, HY-K1082). Then RNA quality was analyzed by the 2100 Bioanalyser (Agilent) and quantified using the ND-2000 (NanoDrop Technologies). Only high-quality RNA sample (OD260/280 = 1.8∼2.2, OD260/230 ≥ 2.0, RIN ≥ 6.5, 28S:18S ≥ 1.0, >1 μg) was used to construct the sequencing library.

### Library preparation, sequencing, and processing

RNA-sequencing transcriptome libraries were prepared following the TruSeq™ RNA sample preparation Kit from Illumina using 1 μg of total RNA. Messenger RNA was isolated according to the polyA selection method by oligo(dT) beads and then fragmented by fragmentation buffer. Next, double-stranded cDNAs were synthesized using a SuperScript double-stranded cDNA synthesis kit (Invitrogen, CA) with random hexamer primers (Illumina). The synthesized cDNAs were subjected to end-repair, phosphorylation, and “A” base addition according to the Illumina’s library construction protocol. Libraries were size selected for cDNA target fragments of 300 bps on 2% Low Range Ultra Agarose followed by PCR amplified using the Phusion DNA polymerase (NEB) for 15 PCR cycles. After quantified by TBS380, paired-end RNA-seq sequencing library was sequenced with the Illumina HiSeq xten/NovaSeq 6000 sequencer (2X150 bp read length). The raw paired end reads were trimmed and quality controlled by SeqPrep and Sickle (Pertea et al., 2015). The original data after quality control, namely clean data (reads), were compared with the reference genome to obtain mapped data (reads) for subsequent transcriptome assembly and expression calculation, etc. At the same time, the quality of the comparison results of this transcriptome sequencing was evaluated, software used: HiSat2, TopHat2 (Kim et al., 2015).

### Differential expression analysis

In RNA-Seq analysis, gene expression levels were calculated by the number of clean reads (reads counts) located to genomic regions. RSEM software was used to quantitatively analyze the expression levels of genes and transcripts, so as to analyze the differential expression (DE) of genes among different samples (Li and Dewey, 2011). DESeq2 software is used to analyze the DE, according to the comparison to the calculated DEGs read count data. The default screening criteria for significantly DEGs were adjusted using *p*-value < 0.05 and log2FC ≧ 1, when a gene met these two conditions at the same time, it was considered as a DEG (Love et al., 2014).

### Pathway analysis

With the GO database, genes can be grouped by their function according to the biological processes (BP), cellular component (CC), and molecular function (MF). The DEGs were annotated with GO, and the results were mapped with up-down-regulated gene GO annotation histogram, using software Blast2go (Ding et al., 2018; Liu and Thomas, 2019). Using the KEGG database, genes can be classified according to the pathway they participate. KEGG annotation of DEGs can display the differential genes on the KEGG pathway map, and display the KEGG annotation pathway maps of up-regulated and down-regulated differential genes, using software KOBAS (Kanehisa et al., 2016). Using the EggNOG database, genes can be classified according to their function. COG annotation was made for DEGs displayed on the COG classification statistical map, using the software BLAST+, Diamond (Li et al., 2018).

### Reverse transcription quantitative PCR

Quantitative PCR experiments were performed as described (Croset et al., 2018). Total RNAs were extracted from the sorted glia and reverse-transcribed using the HiScript III RT SuperMix for qPCR (+gDNA wiper) (vazyme, R323-01). qPCR was performed in AB Quanut Studio 7 Real-Time PCR System using ChamQ Universal SYBR qPCR Master Mix (vazyme, Q711-02). Student’s *t*-test was used to statistic differences between control group and treated group, ^ns^*p* > 0.05; *p < 0.05, ^**^p < 0.01, ^***^p < 0.001, ^****^p < 0.0001.

### Calcium imaging

Calcium imaging experiments were performed using adult flies of 3–5 days old (Owald et al., 2015; Jacob and Waddell, 2020). Flies were put on ice for immobilization, then glued into the dish. We used an external saline containing 108 mM NaCl, 5 mM KCl, 2 mM CaCl_2_⋅2H_2_O, 8 mM MgCl_2_⋅6H_2_O, 4 mM NaHCO_3_, 1 mM NaH_2_PO_4_, 1 mM NaH_2_PO_4_⋅2H_2_O, 10 mM glucose, 5 mM treharose⋅2H_2_O, 10 mM sucrose, 5 mM HEPES, and 1 μM TTX. The fly heads were opened under room temperature in external saline solution. Fluorescence microscope with high frame rate camera was used to record the calcium signals. Images were taken every 40 ms lasting 2 min, and a 10 mM concentration of neurotransmitter was added at 30 s for 5 s. Manual selection and Fiji was used for further analysis (Schindelin et al., 2012). ΔF/F was calculated by fluorescence after addition of neurotransmitter subtracting the baseline fluorescence level (Tian et al., 2009; Vicario and Cali, 2019). To eliminate for baseline differences in F/F0 between samples, we normalized the “preconditioning” treatment for each cell to equal 0 (Park et al., 2022).

## Data availability statement

The data presented in the study are deposited in the NCBI repository, accession number PRJNA992962.

## Ethics statement

The manuscript presents research on animals that do not require ethical approval for their study.

## Author contributions

MW and MH conceived and designed the study, analyzed the data, and wrote the manuscript. MW performed the experiments. Both authors read and approved the manuscript.

## Supplementary material

Supplementary Figure 1 Reverse transcription quantitative PCR verification of common candidate genes selected from the RNA sequencing analysis **(A–C)** RT-qPCR verification of common candidate genes related to signal transduction **(A)**, glial activity **(B)**, and metabolism **(C)**. Common genes include: *nAChRα5*, *nAChRα6*, *nAChRα7*, *Gat*, and *GluRIIA* (receptors and transporters); *Tret1-1*, *Treh*, *ldgf3*, *ldgf4*, and *GS2* (metabolism). ns: *p* > 0.05; *: *p* < 0.05, ^**^: *p* < 0.01, ^***^: *p* < 0.001, ^****^: *p* < 0.0001.

Supplementary Figure 2 Reverse transcription quantitative PCR verification of specific candidate genes in different groups selected from the RNA sequencing analysis **(A–E)** candidate genes were selected from the RNA-sequencing results and their expression changes were verified again using RT-qPCR. Genes selected include: *Fdh*, *Gat1*, *Got*, *mGluR*, *Eaat1*, and pdgy (receptors and transporters); *ldgf2*, *ldgf6* (metabolism). ns: *p* > 0.05; *: *p* < 0.05, ^**^: *p* < 0.01, ^***^: *p* < 0.001, ^****^: *p* < 0.0001.

Supplementary Figure 3 COG classification of RNA-sequencing analysis on glia treated with different types of neurotransmitters **(A–E)** The COG classification of RNA-sequencing results from glia treated with ACh **(A)**, ATP **(B)**, DA **(C)**, GABA **(D)**, and Glu **(E)**.

Supplementary Figure 4 GO annotation of RNA-sequencing analysis on glia treated with different types of neurotransmitters. **(A–E)** The GO annotation of RNA-sequencing results from glia treated with ACh **(A)**, ATP **(B)**, DA **(C)**, GABA **(D)**, and Glu **(E)**.

Supplementary Figure 5 KEGG pathway analysis of RNA-sequencing on glia treated with different types of neurotransmitters. **(A–E)** The KEGG pathway analysis of RNA-sequencing results from glia treated with ACh **(A)**, ATP **(B)**, DA **(C)**, GABA **(D)**, and Glu **(E)**.
