# Supplementary figures and images for: Profiling neurotransmitter-evoked glial responses by RNA-sequencing analysis
Source: Front Neural Circuits. 2023 Aug 14;17:1252759. doi: 10.3389/fncir.2023.1252759 (PMC10461064; doi:10.3389/fncir.2023.1252759)

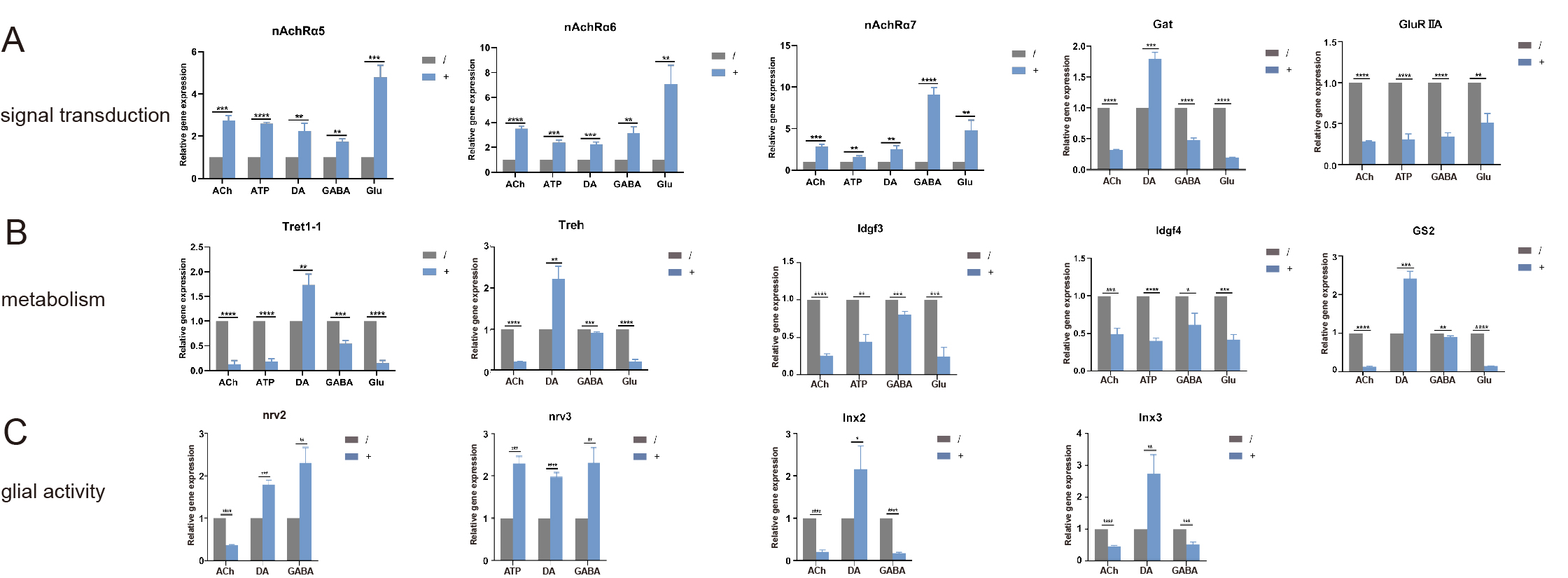

Supplement: Supplementary Figure 1 — Reverse transcription quantitative PCR verification of common candidate genes selected from the RNA sequencing analysis (A–C) RT-qPCR verification of common candidate genes related to signal transduction (A), glial activity (B), and metabolism (C). Common genes include: nAChRα5, nAChRα6, nAChRα7, Gat, and GluRIIA (receptors and transporters); Tret1-1, Treh, ldgf3, ldgf4, and GS2 (metabolism). ns: p > 0.05; *: p < 0.05, **: p < 0.01, ***: p < 0.001, ****: p < 0.0001. [file Image_1.JPEG]

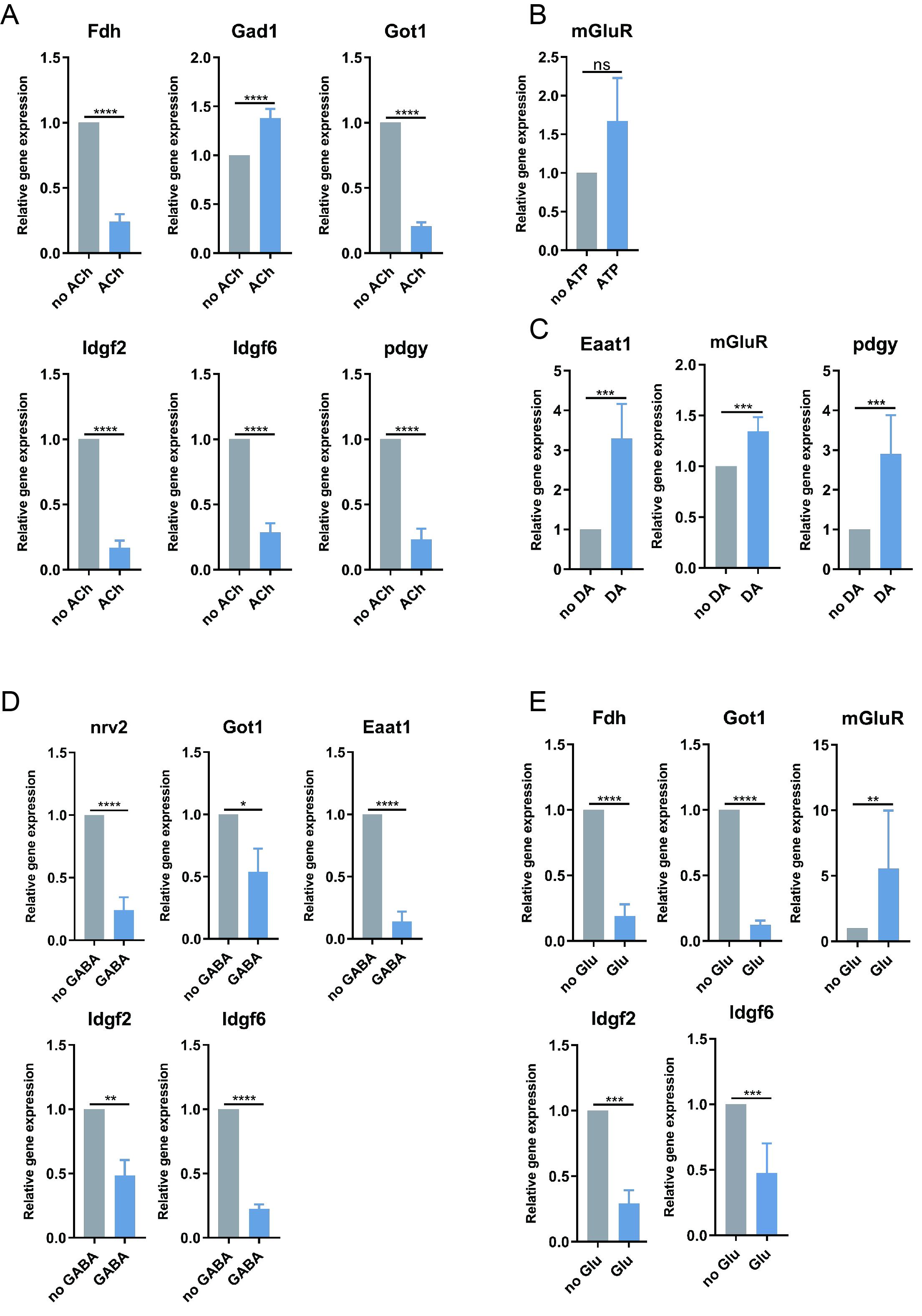

Supplement: Supplementary Figure 2 — Reverse transcription quantitative PCR verification of specific candidate genes in different groups selected from the RNA sequencing analysis (A–E) candidate genes were selected from the RNA-sequencing results and their expression changes were verified again using RT-qPCR. Genes selected include: Fdh, Gat1, Got, mGluR, Eaat1, and pdgy (receptors and transporters); ldgf2, ldgf6 (metabolism). ns: p > 0.05; *: p < 0.05, **: p < 0.01, ***: p < 0.001, ****: p < 0.0001. [file Image_2.JPEG]

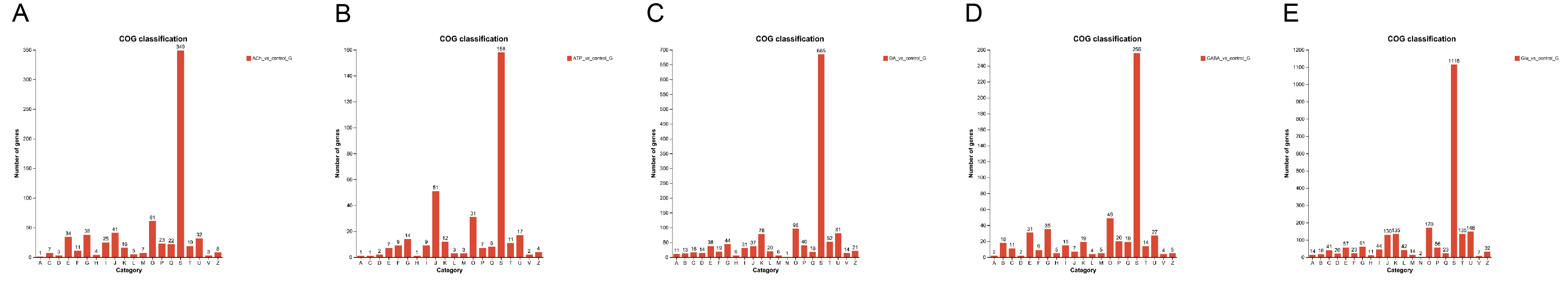

Supplement: Supplementary Figure 3 — COG classification of RNA-sequencing analysis on glia treated with different types of neurotransmitters (A–E) The COG classification of RNA-sequencing results from glia treated with ACh (A), ATP (B), DA (C), GABA (D), and Glu (E). [file Image_3.JPEG]

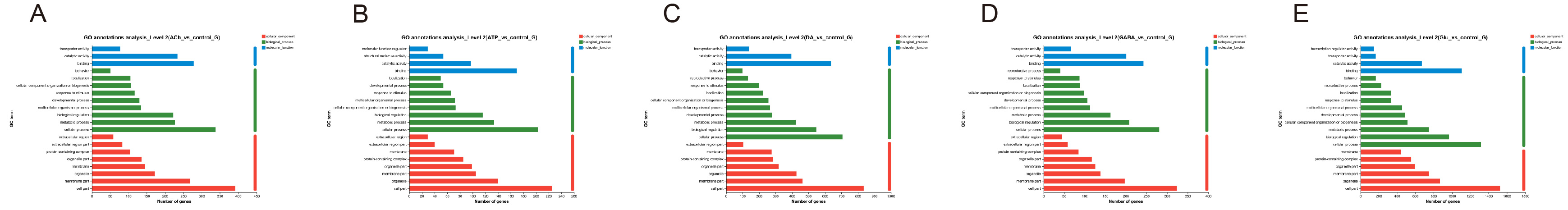

Supplement: Supplementary Figure 4 — GO annotation of RNA-sequencing analysis on glia treated with different types of neurotransmitters. (A–E) The GO annotation of RNA-sequencing results from glia treated with ACh (A), ATP (B), DA (C), GABA (D), and Glu (E). [file Image_4.JPEG]

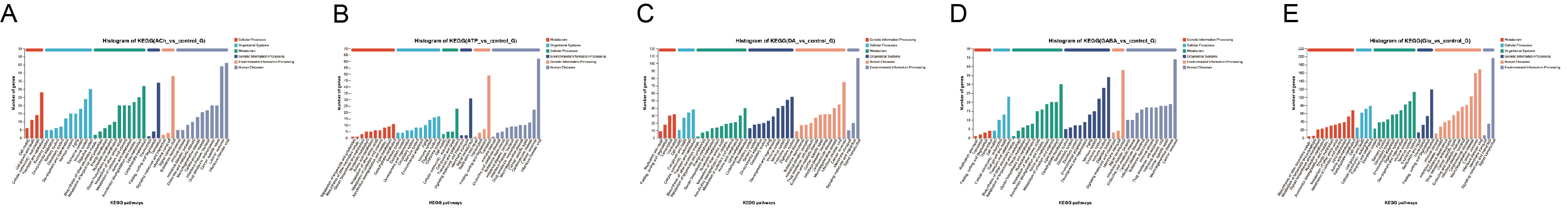

Supplement: Supplementary Figure 5 — KEGG pathway analysis of RNA-sequencing on glia treated with different types of neurotransmitters. (A–E) The KEGG pathway analysis of RNA-sequencing results from glia treated with ACh (A), ATP (B), DA (C), GABA (D), and Glu (E). [file Image_5.JPEG]
